# Supplementary figures and images for: Engineered disulfide reveals structural dynamics of locked SARS-CoV-2 spike
Source: PLoS Pathog. 2022 Jul 29;18(7):e1010583. doi: 10.1371/journal.ppat.1010583 (PMC9365160; doi:10.1371/journal.ppat.1010583)

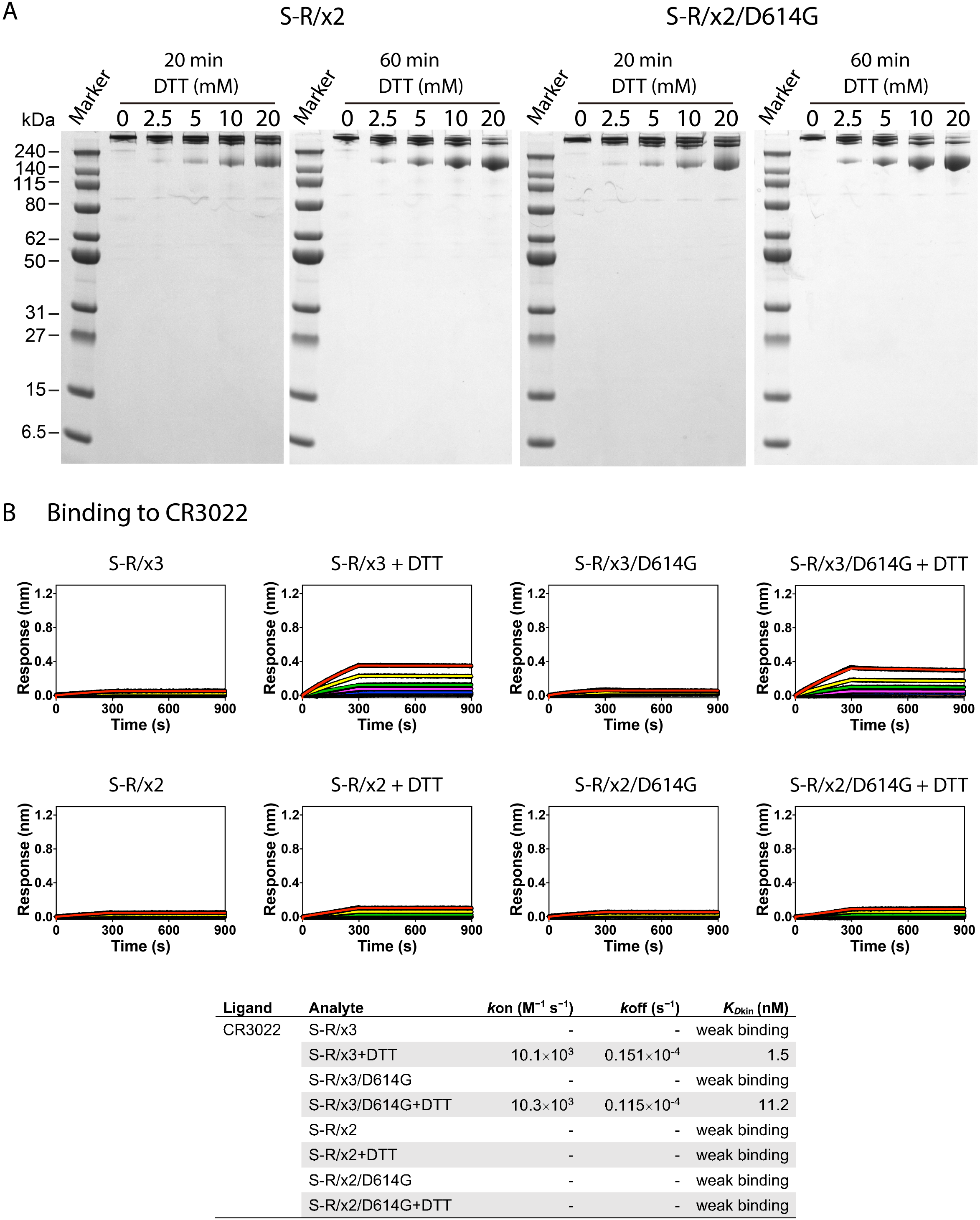

Supplement: S1 Fig — (A) Reduction of x2 disulfide bonds under native conditions by 20 and 60 min incubation with indicated concentrations of DTT, reactions were stopped by excess amount of iodoacetamide before SDS-PAGE. (B) binding of CR3022, an antibody targeting a cryptic epitope on RBD, to different spike proteins in the absence and presence of 20 mM DTT. Spike proteins were serial diluted to 200, 100, 50, 25, 12.5, 6.25, 3.13 and 0 nM and used as analytes in the assay. (TIF) [file ppat.1010583.s001.tif]

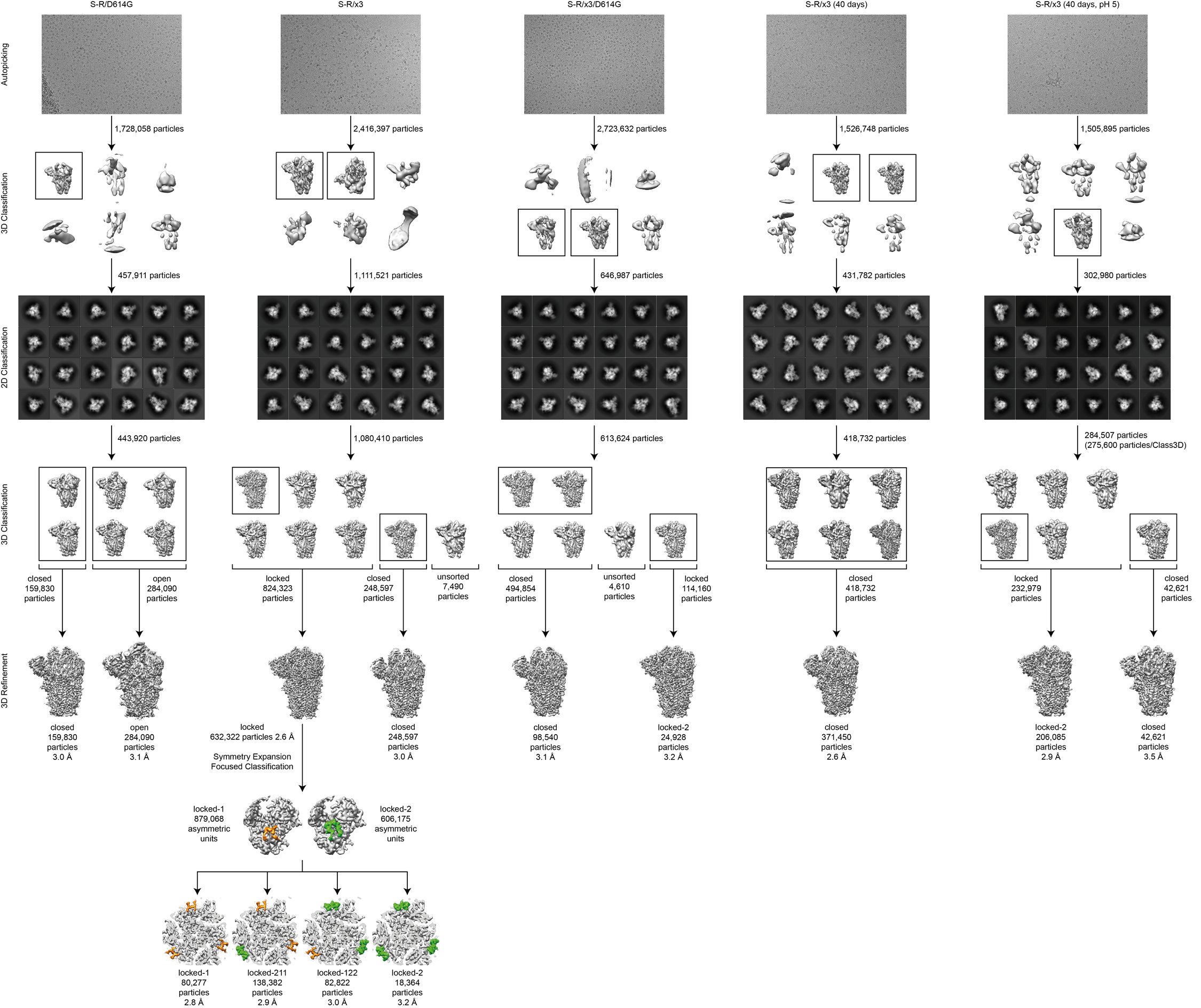

Supplement: S2 Fig — Pipeline is illustrated for S-R/D614G, S-R/x3, S-R/x3/D614G, S-R/x3 after 40 days, and the latter after transfer to pH5.0 buffer. After automated picking, 3D and 2D classification steps were used to remove contaminating objects. 3D classification was then used to sort the data into locked and closed conformations. (TIF) [file ppat.1010583.s002.tif]

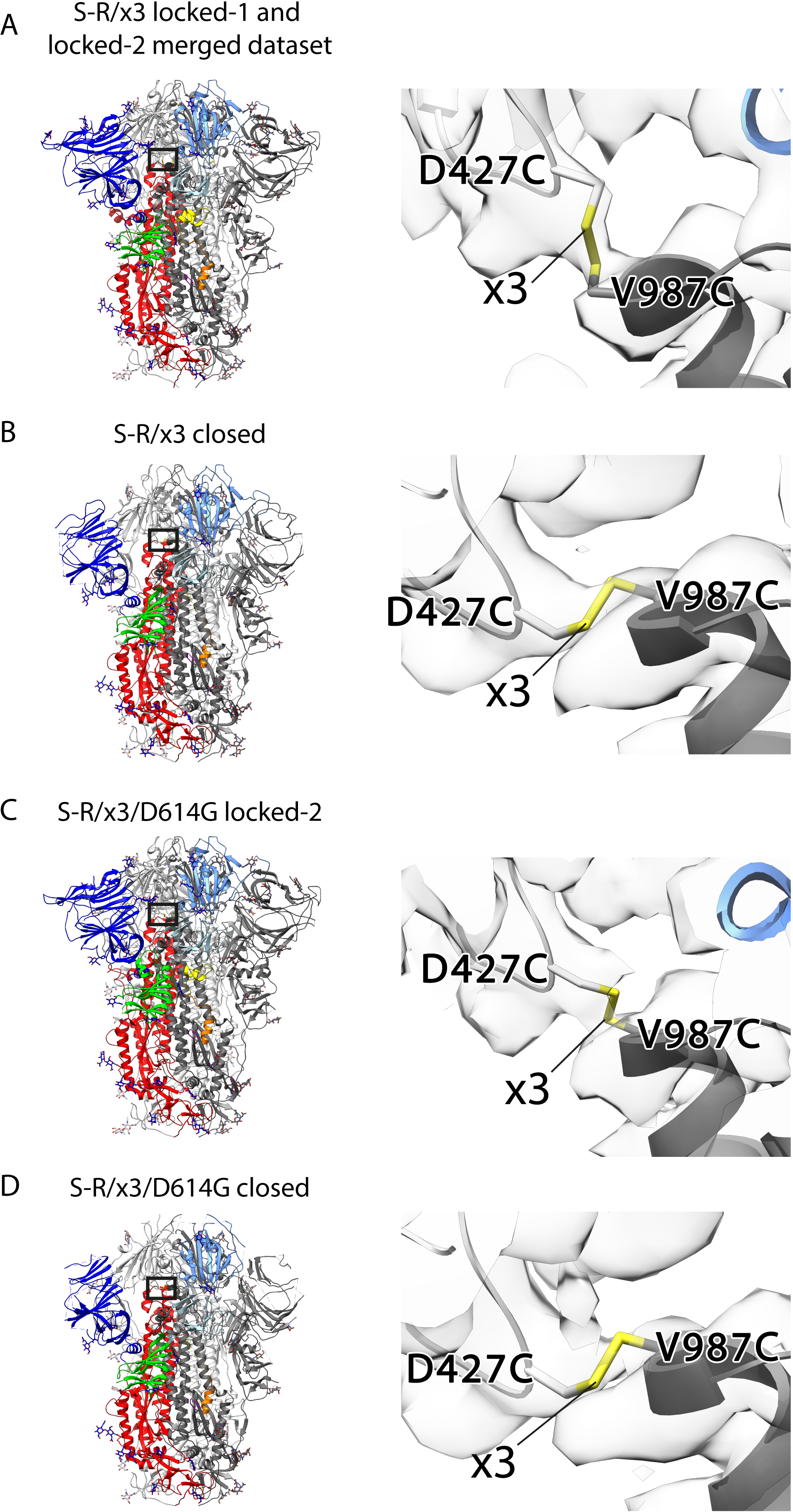

Supplement: S3 Fig — (A)—(D), left panels show the locations of the x3 disulfide bonds within the corresponding spike trimer structures; right panels show molecular models and densities of the x3 disulfide bonds. The radiation damage caused fading of the disulphide bond between the two engineered cysteines in the S-R/x3 and S-R/x3/D614G final maps. In order to recover the disulfide bond densities, EM maps of closed and locked S trimers were reconstructed from the first 4 frames in the movies of S-R/x3 and the first 5 frames of S-R/x3/D614G, respectively. Distinct densities of the disulfide bond were observed in the EM structures reconstructed from early exposed frames and are shown the right panels. (TIF) [file ppat.1010583.s003.tif]

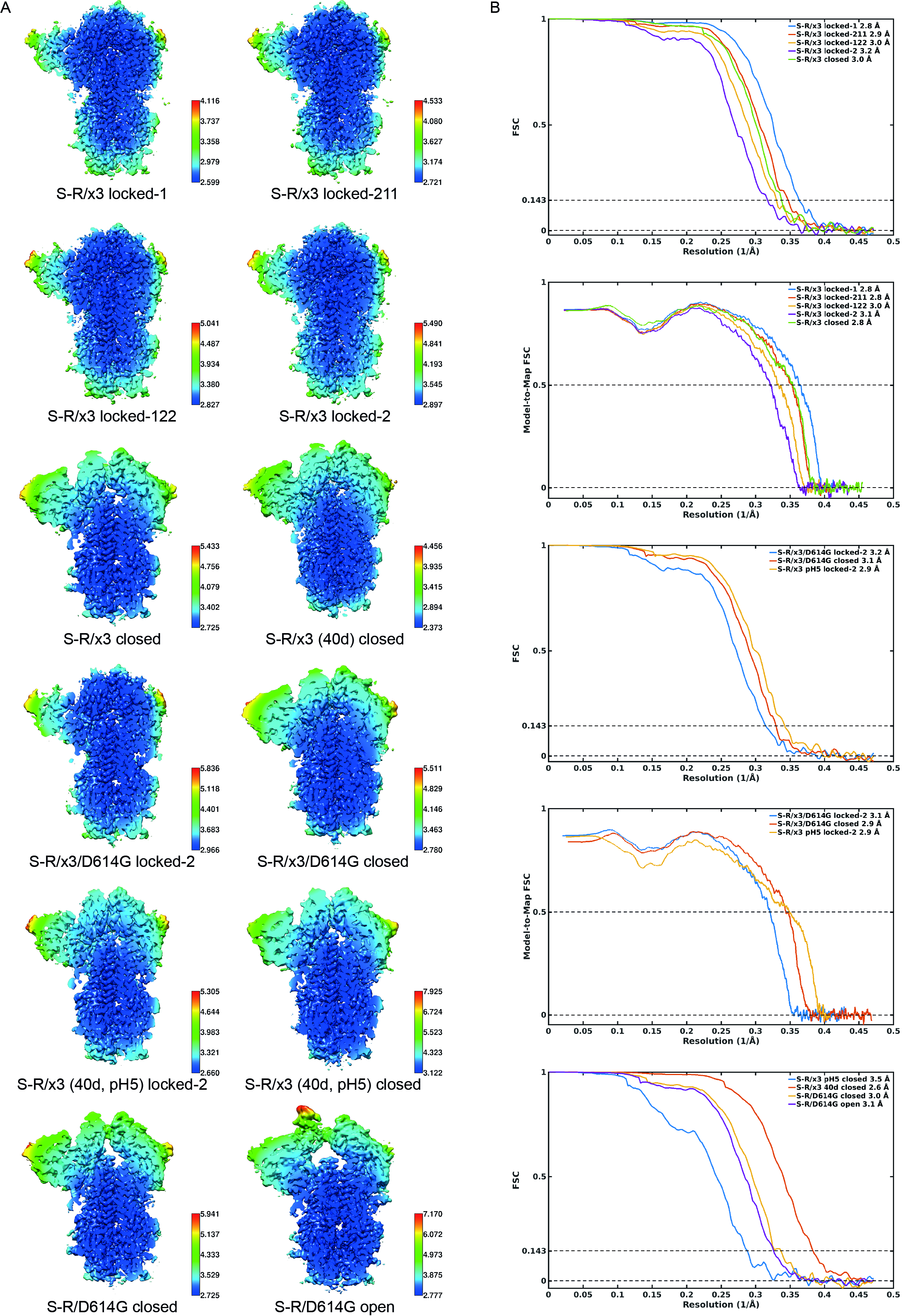

Supplement: S4 Fig — (A) Local resolution maps for all structures calculated using RELION. (B) Global resolution assessment by Fourier shell correlation at the 0.143 criterion (left panel), and correlations of model vs map by Fourier shell correlation at the 0.5 criterion (right panel). (TIF) [file ppat.1010583.s004.tif]

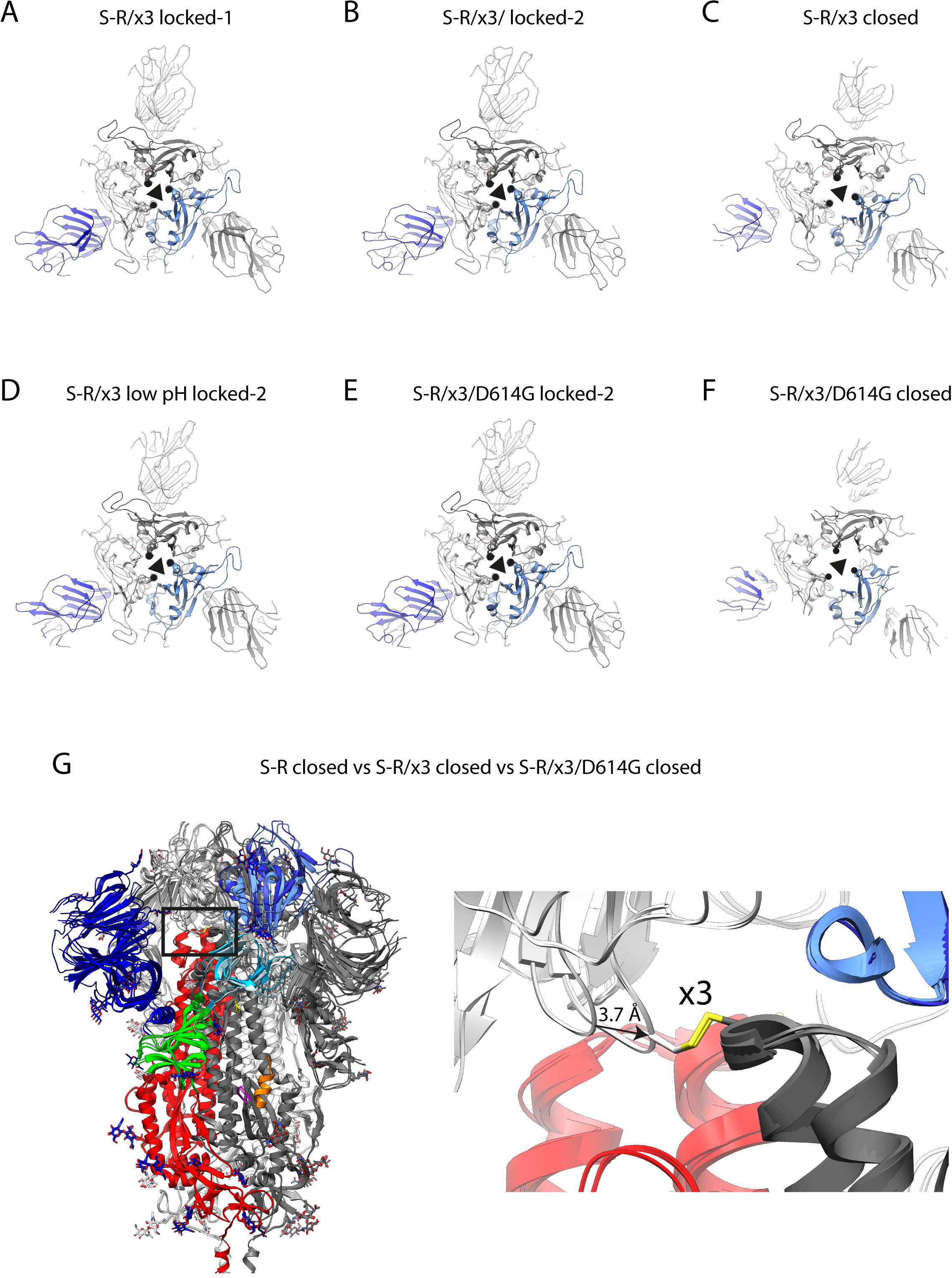

Supplement: S5 Fig — (A)—(F), top views of S-R/x3 and S-R/x3/D614G spikes in locked-1, locked-2 and closed conformational states. The three-fold axes are indicated by triangles. The structural elements that are closest to the three-fold axes in the locked structures are indicated by dots (panels (A), (B), (D), (E)). The same structural elements are indicated by dots in the closed conformations to highlight the movement of the receptor binding domains between the two conformational states (panels (C) and (F)). (G) comparison of S-R, S-R/x3 and S-R/x3/D614G spikes in closed conformations. Left panel, overlay of the three structures, the box indicates the zoomed-in view in the right panel. Right panel, a movement of 3.7 Å was observed between the closed S-R (without x3 disulphide) and the closed S-R/x3 and S-R/x3/D614G spikes (with x3 disulphide). (TIF) [file ppat.1010583.s005.tif]

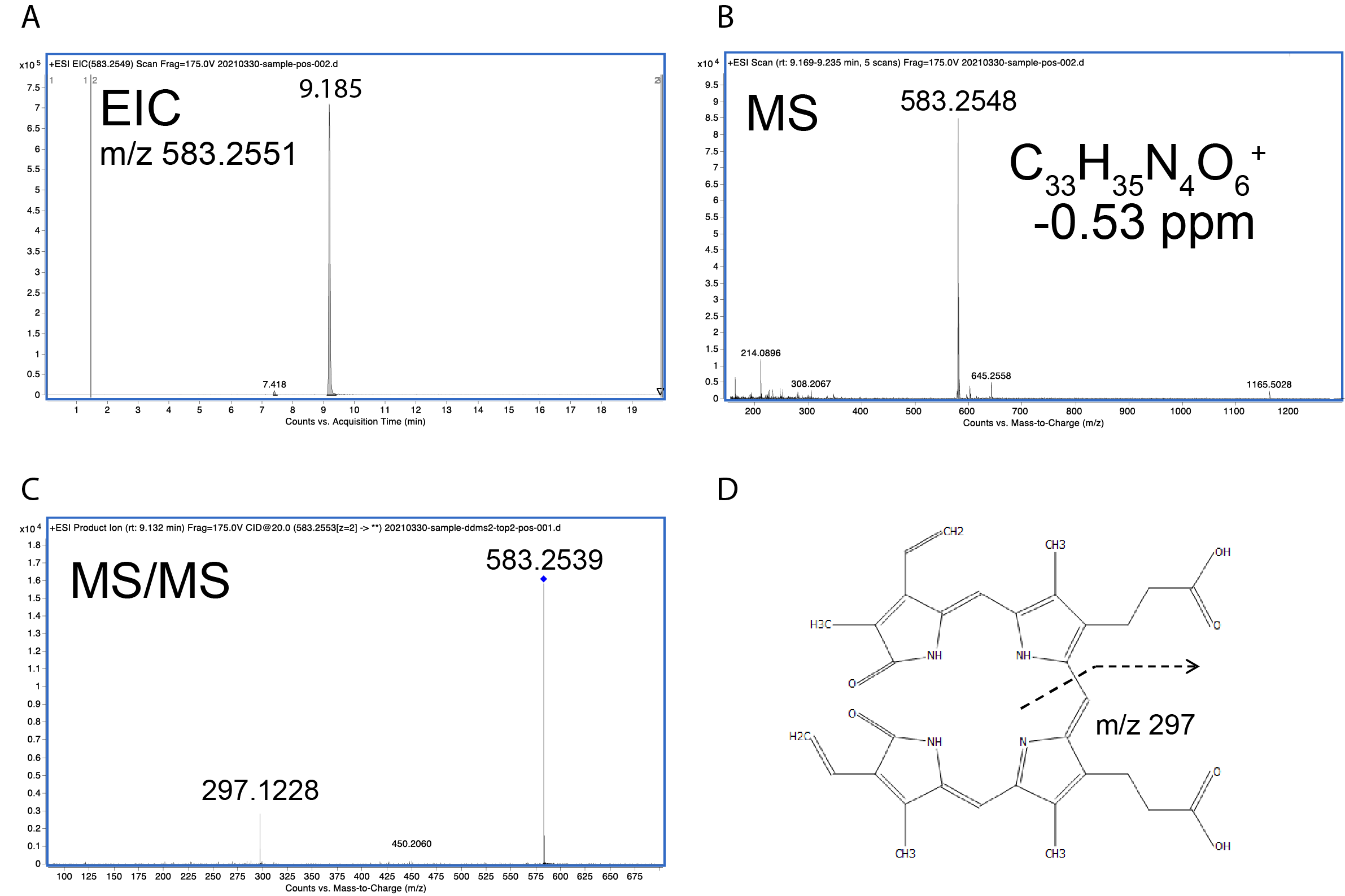

Supplement: S6 Fig — (A) extracted-ion chromatogram (EIC) of organic extract of purified spike protein identifies a prominent peak with an elution time of 9.185 min. (B) MS analysis of the peak in panel a identifies a major ion species with an m/z of 583.2551 consistent to the structure of biliverdin IXα (panel (D)) with a mass difference of -0.53 ppm. (C) MS/MS spectrum of biliverdin IXα in the sample identifies the parent ion (indicated by the blue diamond) and a fragment with an m/z of 297.1228 corresponding to the proposed fragment ion of biliverdin IXα (indicated by the dashed line in panel (D)). (D) Chemical structure of biliverdin Ixα and proposed fragmentation pattern in MS/MS (dashed line). (TIF) [file ppat.1010583.s006.tif]

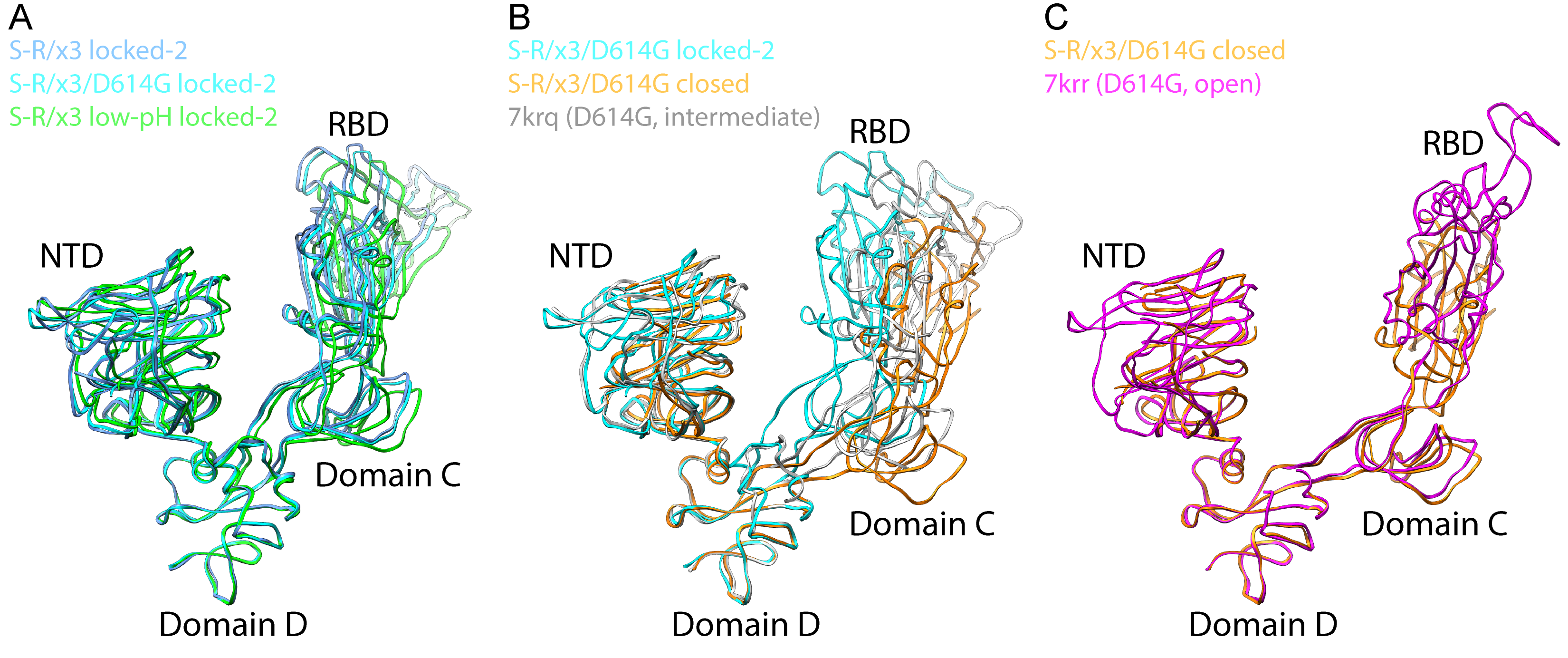

Supplement: S7 Fig — (A)—(C), S1 parts of various different spike structures were aligned using domain D to highlight the movement of the RBD and domain C between the different structures. (TIF) [file ppat.1010583.s007.tif]

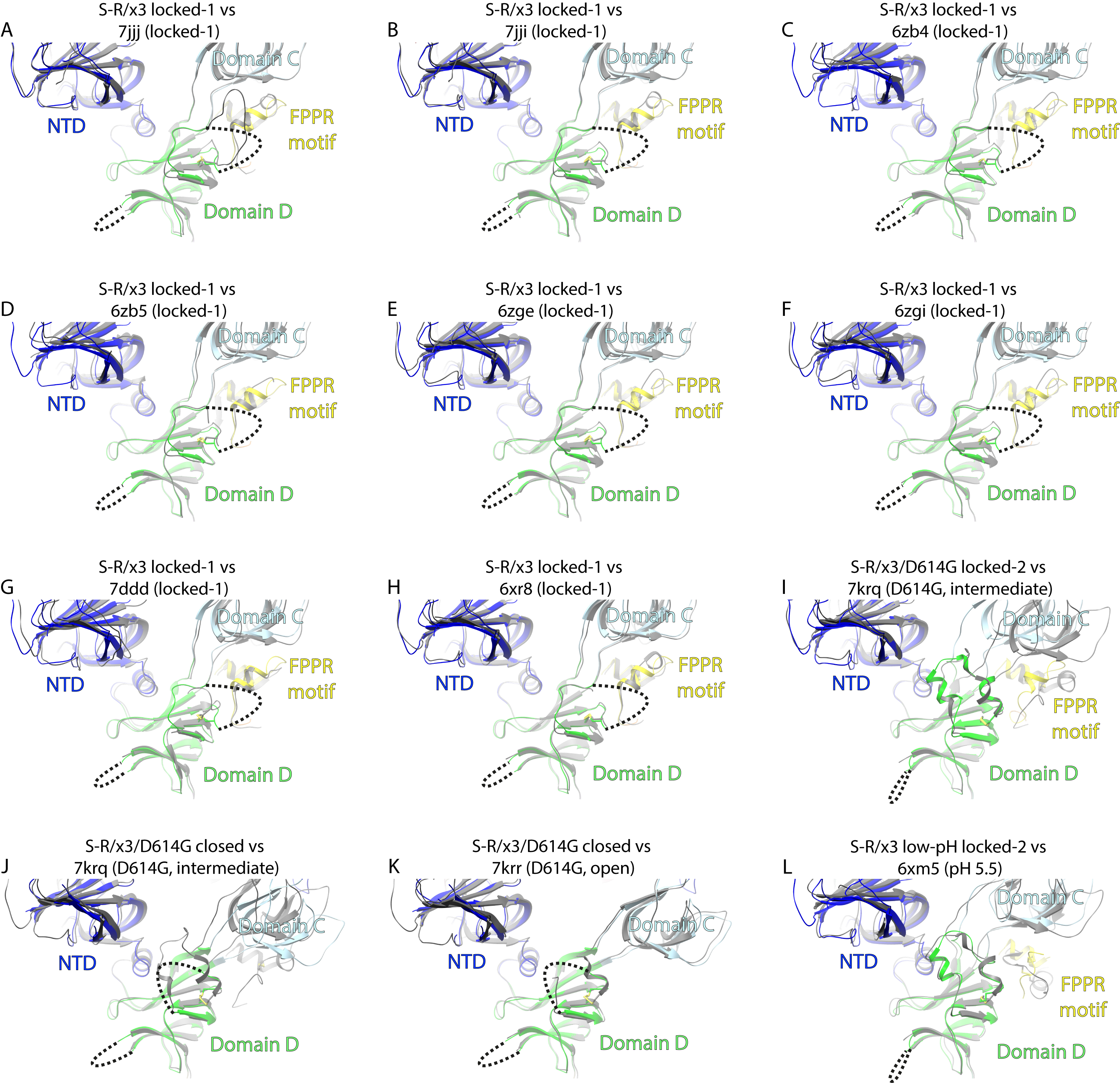

Supplement: S8 Fig — (A)—(H), S-R/x3 locked-1 (colored) was compared to various locked SARS-CoV-2 spike structures (gray), in most of these structures, the domain D and surrounding area adopt a similar structure as in the S-R/x3 locked-1 structure. (I)—(J), The full-length D614G SARS-CoV-2 spike structure (gray, PDB: 7krq) was compared to S-R/x3/D614G locked-2 and S-R/x3/D614G closed structures (colored) to show the structure is in a intermediate state between locked and closed conformations. (K) S-R/x3/D614G closed structure compared to the full-length D614G SARS-CoV-2 spike in open conformation. (L) S-R/x3 low-pH locked-2 structure compared to another deposited low-pH SARS-CoV-2 spike structure (6xm5, chain A), which shows the most well-defined densities for regions around domain D among their low-pH structures (6xm4, 6xm5, 6xlu, 7jwy) [23]. PDB-IDs are from the following studies: 7jjj, 7iii [8]; 6zb4, 6zb5 [10]; 6zge, 6zgi [21]; 7ddd [14]; 6xr8 [9]; 7krq, 7qrr [22]; 6xm5 [23]. (TIF) [file ppat.1010583.s008.tif]
