# Supplementary material for: Engineered disulfide reveals structural dynamics of locked SARS-CoV-2 spike
Source: PLoS Pathog. 2022 Jul 29;18(7):e1010583. doi: 10.1371/journal.ppat.1010583 (PMC9365160; doi:10.1371/journal.ppat.1010583)
Supplement: S1 Table — (PDF) [file ppat.1010583.s009.pdf]

S1 Table. Cryo-EM data collection, refinement and validation statistics

	S-R/x3 locked-1 (EMD-33453, PDB 7xtz)	S-R/x3 locked-211 (EMD-33454, PDB 7xu0)	S-R/x3 locked-122 (EMD-33455, PDB 7xu1)	S-R/x3 locked-2 (EMD-33456, PDB 7xu2)	S-R/x3 closed (EMD-33457, PDB 7xu3)
**Data collection and processing**					
Magnification			81,000		
Voltage (kV)			300		
Electron exposure (e⁻/Å²)			50		
Defocus range (μm)			1.0-2.4		
Pixel size (Å)			1.061		
Movies (no.)			6,642		
Initial particle images (no.)			2,416,397		
Symmetry imposed	*C3*	*C1*	*C1*	*C3*	*C3*
Final particle images (no.)	80,277	138,382	82,822	18,364	248,597
Map resolution (Å)	2.8	2.9	3.0	3.2	3.0
FSC threshold	0.143	0.143	0.143	0.143	0.143
Map resolution range (Å)	2.60-5.50	2.72-5.86	2.83-6.93	2.90-7.35	2.73-6.69
**Refinement**					
Initial model used		PDB 6zp2			PDB 6zox
Model resolution (Å)	2.8	2.8	3.0	3.1	2.8
FSC threshold	0.5	0.5	0.5	0.5	0.5
Map sharpening *B* factor (Å²)	-40	-40	-40	-40	-25.3
Model composition					
Non-hydrogen atoms	26871	26898	26926	26979	24240
Protein residues	3291	3300	3309	3321	3006
Ligands	75	72	69	66	48
B factors (Å²)					
Protein	48.15	51.56	59.89	68.03	92.77
Ligand	66.34	68.92	81.72	84.37	103.53
R.m.s. deviations					
Bond lengths (Å)	0.004	0.004	0.004	0.005	0.006
Bond angles (°)	1.001	0.953	1.021	1.083	0.836
**Validation**					
MolProbity score	1.25	1.36	1.46	1.53	1.43
Clashscore	2.62	3.88	4.11	3.63	3.73
Poor rotamers (%)	0.42	0.14	0.24	0.86	0.34
Ramachandran plot					
Favored (%)	96.76	96.89	96.16	94.45	96.13
Allowed (%)	3.24	3.11	3.84	5.55	3.87
Disallowed (%)	0	0	0	0	0

	S-R/x3/D614G locked-2 (EMD-33458, PDB 7xu4)	S-R/x3/D614G closed (EMD-33459, PDB 7xu5)	S-R/D614G closed (EMD-33463)	S-R/D614G open (EMD-33464)
**Data collection and processing**				
Magnification	81,000		81,000	
Voltage (kV)	300		300	
Electron exposure (e⁻/Å^2)	50		50	
Defocus range (μm)	1.0-2.4		1.0-2.4	
Pixel size (Å)	1.061		1.061	
Movies (no.)	3,036		1,980	
Initial particle images (no.)	2,723,632		1,728,058	
Symmetry imposed	*C3*	*C3*	*C3*	*C1*
Final particle images (no.)	24,928	98,540	159,830	284,090
Map resolution (Å)	3.2	3.1	3.0	3.1
FSC threshold	0.143	0.143	0.143	0.143
Map resolution range (Å)	2.97-7.94	2.78-6.78	2.73-7.28	2.78-9.88
**Refinement**				
Initial model used	PDB 6zoz	PDB 6zox		
Model resolution (Å)	3.2	2.9		
FSC threshold	0.5	0.5		
Map sharpening *B* factor (Å^2)	-30.0	-30.0	-40	-40
Model composition				
Non-hydrogen atoms	27063	22755		
Protein residues	3330	2814		
Ligands	69	45		
B factors (Å^2)				
Protein	77.74	76.80		
Ligand	93.88	88.66		
R.m.s. deviations				
Bond lengths (Å)	0.005	0.006		
Bond angles (°)	1.096	0.709		
**Validation**				
MolProbity score	1.64	1.35		
Clashscore	4.50	2.92		
Poor rotamers (%)	0.31	0		
Ramachandran plot				
Favored (%)	93.72	96.11		
Allowed (%)	6.28	3.89		
Disallowed (%)	0	0		

	S-R/x3 (40d) closed (EMD-33461)	S-R/x3 (40d, pH5) locked-2 (EMD-33460, PDB-7xu6)	S-R/x3 (40d, pH5) closed (EMD-33462)
**Data collection and processing**			
Magnification	81,000	81,000	
Voltage (kV)	300	300	
Electron exposure (e^{-}/Å^2)	50	50	
Defocus range (μm)	1.0-2.4	1.0-2.4	
Pixel size (Å)	1.061	1.061	
Movies (no.)	1,839	1,698	
Initial particle images (no.)	1,526,748	1,505,895	
Symmetry imposed	*C3*	*C3*	*C3*
Final particle images (no.)	371,450	232,979	42,621
Map resolution (Å)	2.6	2.9	3.5
FSC threshold	0.143	0.143	0.143
Map resolution range (Å)	2.37-5.47	2.66-6.73	3.12-9.79
**Refinement**			
Initial model used		PDB 6zoz	
Model resolution (Å)		2.9	
FSC threshold		0.5	
Map sharpening *B* factor (Å^2)	-31.9	-21.8	-50
Model composition			
Non-hydrogen atoms		26565	
Protein residues		3267	
Ligands		66	
B factors (Å^2)			
Protein		92.97	
Ligand		110.52	
R.m.s. deviations			
Bond lengths (Å)		0.005	
Bond angles (°)		1.099	
**Validation**			
MolProbity score		1.71	
Clashscore		5.18	
Poor rotamers (%)		0.74	
Ramachandran plot			
Favored (%)		93.22	
Allowed (%)		6.78	
Disallowed (%)		0	
