# Supplementary material for: Engineered disulfide reveals structural dynamics of locked SARS-CoV-2 spike
Source: PLoS Pathog. 2022 Jul 29;18(7):e1010583. doi: 10.1371/journal.ppat.1010583 (PMC9365160; doi:10.1371/journal.ppat.1010583)
Supplement: Attachment — Submitted filename: D614G_ResponseToReviewers_220419.pdf [file ppat.1010583.s010.pdf]

We thank the reviewers for their careful and constructive consideration of our manuscript.

Part I - Summary
Please use this section to discuss strengths/weaknesses of study, novelty/significance, general execution and scholarship.

Reviewer #1: The manuscript from Qu et al investigates the locked conformation of the SARS-CoV-2 spike, a conformation that is infrequently observed by cryo-EM studies of spike protein as compared to the closed or open conformations. The locked conformation has not been observed in high-resolution structures of spikes on virions, however, raising some concern as to whether the locked conformation is an in vitro artifact. To more easily study the locked conformation, the authors design a disulfide bond by substitution D427 and V987 with cysteine. Biochemical characterization, including non-reducing SDS-PAGE, confirms that the disulfide bond does form as intended and prevents ACE2 binding, as expected. Cryo-EM analysis of this variant shows that 76% of the particles are in the locked conformation and 23% are in the closed conformation. Data processing is able to discriminate two different locked conformations, named locked-1 and locked-2, that differ primarily by the position of residues in domain D. Introduction of the D614G substitution shifts the distribution of spikes toward the closed conformation, suggesting that D614G makes the spikes inherently less stable and more prone to open, which is consistent with data and conclusions published in 2020.

The manuscript has several strengths. The design of a disulfide-stabilized locked spike, or mostly locked spike, is interesting, and may be of use to the field. The biochemical and structural characterizations are very thorough and are performed to a high standard. The writing, although technical in nature, is clear. The major weakness or limitation of the manuscript is the potential lack of physiological relevance. As noted by the authors and mentioned above, the locked state has not been observed in structures of spikes on the surface of virions. A potential explanation is that the locked state is some artifact of recombinantly expressed and purified spike protein. The authors primarily speculate about the functional role of the locked-1 and locked-2 states (lines 359 and 387), but do not experimentally support their claims. And even if the locked state is sampled by spikes on infectious virions in vivo, it is possible that the different locked-1 and locked-2 states observed in these studies is due to the artificial nature of the introduced disulfide bond. Overall, it is solid work, but may be too preliminary at this time.

We would like to briefly respond to the reviewer's concern that the locked structures may lack physiological relevance. The x3 crosslink is not close to regions which fold in the locked states, and the crosslink leaves sufficient conformational flexibility to allow both locked conformations and the closed conformation to form. In the section "Comparison to other spike structures" we described that the locked-1 conformation has been observed in purified S protein under multiple different conditions without the introduction of a disulphide bond. We also suggest why the locked-2 conformation has not previously been observed – in the absence of stabilizing mutations, the D614G spike (where the locked-2 conformation is preferentially observed) transitions to the closed and open forms. This effect is exacerbated by use of a double proline mutation in many studies which further stabilizes the open state (we have rephrased this part of the text in an effort to make this point more clearly). The absence of the locked state on the purified virus is entirely consistent with our model that the locked state is adopted transiently during assembly. While we cannot formally rule out the possibility that the locked conformations are artefacts of working with purified protein, we consider this highly unlikely.

Reviewer #2: Investigators have advanced prior studies of SARS-CoV-2 spike structures by "locking" spike ectodomains with an engineered (aa 427 to aa 987) disulfide bridge. Biochemical tests revealed the disulfide locked ectodomains did not display RBDs to ACE2 (RBD-down) but RBDs could be raised for ACE2 binding by DTT reduction. Cryo-EM showed that most disulfide locked trimers were in closed conformations. Further analysis revealed two distinct locked conformations. The D614G change reduced the proportion of locked conformations. These are important results that advance understanding of spike protein structures in different conformations.

Prolonged incubation of the locked trimers resulted in conversion to a "closed" conformation, and then exposure of the closed form to pH5 re-locked the trimers. These changes regulating locked to closed transitions involved specific subdomains, notably the NTD associated domains. The manuscript includes impressive detail on the structural differences between the locked and closed conformations, and the effects of a D614G substitution on these differences.

Structures are used to infer models of spike dynamics during virus secretion. The suggestion is that virus spikes are assembled into locked conformations during virus assembly and egress. This is based on the assumption that viruses egress through acidic organelles, which lock the trimers. Once secreted, virus spikes at neutral pH slowly convert to closed conformations, which are prerequisite to RBD opening.

The models are very intriguing, however, it is recommended that they be further considered. The results of Ghosh et al. indicate that organelles through which the viruses egress are at neutral pH – there are accessory proteins of the virus that neutralize endolysosome lumenal compartments. Furthermore, the natural spikes undergo furin cleavage in the TGN, which effects conformational changes that were not described in the models. In addition, the D614G change that is highlighted in the model is known to provide a stabilizing force, maintaining the S1-S2 heterodimers. It is difficult to appreciate this important S1-S2 stabilizing property in the context of the model involving locked and closed conformations. Finally, the notion that there are very slow transitions from the locked to closed, and then to open conformations of spike do not appear consistent with the very rapid cell membrane fusions that take place when spike-expressing cells are incubated with ACE2-expressing target cells. In this case, as soon as spikes appear on plasma membranes, they promote cell membrane fusions, suggesting that they are in open conformations.

Part II – Major Issues: Key Experiments Required for Acceptance
Please use this section to detail the key new experiments or modifications of existing experiments that should be absolutely required to validate study conclusions.

Generally, there should be no more than 3 such required experiments or major modifications for a "Major Revision" recommendation. If more than 3 experiments are necessary to validate the study conclusions, then you are encouraged to recommend "Reject".

Reviewer #1: (No Response)

Reviewer #2: The models are very intriguing, however, it is recommended that they be further considered. [point 1: The results of Ghosh et al. indicate that organelles through which the viruses egress are at neutral pH – there are accessory proteins of the virus that neutralize endolysosome lumenal compartments.] [point 2: Furthermore, the natural spikes undergo furin cleavage in the TGN, which effects conformational changes that were not described in the models.] [point 3: In addition, the D614G change that is highlighted in the model is known to provide a stabilizing force, maintaining the S1-S2 heterodimers. It is difficult to appreciate this important S1-S2 stabilizing property in the context of the model involving locked and closed conformations.] [point 4: Finally, the notion that there are very slow transitions from the locked to closed, and then to open conformations of spike do not appear consistent with the very rapid cell membrane fusions that take place when spike-expressing cells are incubated with ACE2-expressing target cells. In this case, as soon as spikes appear on plasma membranes, they promote cell membrane fusions, suggesting that they are in open conformations.]

Point 1: Ghosh et al do indeed show that accessory proteins deacidify the lysosomes through which the egressing virus passes but they also show that these compartments nevertheless remain acidic. We have slightly rephrased the sentence referring to this paper: "*Beta-coronaviruses have recently been shown to assemble in the low pH, high lipid environment of the ERGIC, and to egress through somewhat acidic compartments of the endomembrane system that are adapted by viral accessory proteins (Ghosh et al., 2020)*".

Point 2 and 3: Our study was specifically designed to address the structure and function of locked conformations of the spike. We have not explored any stabilizing effects of the D614G mutation on the open conformation, nor have we explored conformational effects promoted by furin cleavage. There is extensive published data indicating that the D614G mutation promotes the open conformation of the spike, and our study of the locked conformation provide one explanation for this observation. There is also good data that the open conformation of the spike is stabilized by the D614G mutation, that this mutation may reduce shedding, and that furin cleavage can also promote opening of the spike. Such effects would be in addition to our model. We have tried to clarify these points in three ways:

First, we have made phrasing changes the text to reduce possible confusion between factors that may increase the stability of a particular conformation of S, and factors that may increase the overall stability of the S protein.

Second, we have edited our discussion of the model, including adding new references.

"*Structural transitions between locked, closed and open conformations are complex, and although we suggest that changes in domain D are a dominant factor in regulating transition between locked and closed conformations, other factors, including linoleic acid binding and furin cleavage, may also play a role.*"

*“The 614D spike may transition from locked-1 to locked-2 to closed to open, while the 614G spike can only adopt the locked-2 conformation, and exhibits different structural dynamics and a higher tendency to open. We suggest that the shifted locked to closed equilibrium results in a higher tendency to open (**Fig. 5a**). This may work in tandem with stabilization of the open conformation by the D614G mutation (Díaz-Salinas et al., 2022; Yang et al., 2021, 2022) or a reduction in spontaneous shedding of the S1 subunit (Jackson et al., 2021; Shi and Xie, 2021).”*

Third, we have edited our schematic model (Fig. 5a) and associated legend to indicate that the closed-open equilibrium may also differ between the 614D and 614G spikes.

Point 4: Regarding the speed of locked to closed to open transitions, we did not intend to imply that these are slow comparable to the timescale of protein expression and secretion in the neutral environment at the cell surface. In the context of spike expressing cells, we would expect much of the cell-surface S protein to be in the open form. We have slightly edited the text in an effort to avoid this misunderstanding: “*Given that both low pH and lipid binding favour locked conformations, we speculate that during virus assembly, both the 614D and 614G spikes are in locked conformations, and that this provides a mechanism to prevent premature transition into open or post-fusion conformations during virus assembly and egress. Once viruses are released into the neutral pH environment outside of the cell, the spike transitions to the closed or open conformations (Fig 5b).*”

Part III – Minor Issues: Editorial and Data Presentation Modifications
Please use this section for editorial suggestions as well as relatively minor modifications of existing data that would enhance clarity.

Reviewer #1: (No Response)

Reviewer #2: (No Response)

Other changes made to the manuscript:

We have corrected a typo in Figure S2 (one of the locked classes was labelled as an open class).

The reference list has been edited to reflect the changes described in the response to reviewers. Please note that changes to referencing are unfortunately not marked in the “tracked” version of the manuscript.
